# The change and the mediating role of parental emotional reactions and depression in the treatment of traumatized youth: results from a randomized controlled study

**DOI:** 10.1186/1753-2000-8-11

**Published:** 2014-04-08

**Authors:** Tonje Holt, Tine K Jensen, Tore Wentzel-Larsen

**Affiliations:** 1 Norwegian Centre for Violence and Traumatic Stress Studies (NKVTS), P.O. Box 181, Nydalen, 0409 Oslo, Norway; 2 Department of Psychology, University of Oslo, P.O. Box 1094, Blindern, 0317 Oslo, Norway; 3 Center for Child and Adolescent Mental Health, Eastern and Southern Norway, P.O.Box 4623, Nydalen, 0405 Oslo, Norway

**Keywords:** Parents, Emotional reactions, Trauma treatment, Children and adolescents

## Abstract

**Background:**

Trauma-Focused Cognitive Behavioral Therapy (TF-CBT) has been shown to efficiently treat children and youth exposed to traumatizing events. However, few studies have looked into mechanisms that may distinguish this treatment from other treatments. The objective of this study was to investigate whether the parents’ emotional reactions and depressive symptoms change over the course of therapy in the treatment conditions of TF-CBT and Therapy as Usual (TAU), and whether changes in the reactions mediate the difference between the treatment conditions on child post-traumatic stress (PTS) symptoms and child depressive symptoms.

**Method:**

A sample of 135 caregivers of 135 traumatized children and youth (*M* age = 14.8, *SD* = 2.2, 80% girls) was randomly assigned to receive either TF-CBT or TAU. The parents’ emotional reactions were measured using the Parental Emotional Reaction Questionnaire (PERQ), and their depressive symptoms were measured using the Center for Epidemiologic Studies Depression Scale (CES-D). The children’s outcomes were post-traumatic stress (PTS) reactions and depression, as measured by the Clinician-Administered PTSD Scale for Children and Adolescents (CAPS-CA) and Mood and Feelings Questionnaire (MFQ), respectively.

**Results:**

The parents’ emotional reactions and depressive symptoms decreased significantly from pre- to post-therapy, but no significant differences between the two treatment conditions were found. The changes in reactions did not significantly mediate the treatment difference between TF-CBT and TAU on child PTS symptoms. However a mediating effect was found on child depressive symptoms.

**Conclusion:**

The results showed that although the parents experienced reductions in emotional reactions and depressive symptoms when their child received therapy, this was only significantly related to the difference in outcome between TF-CBT and TAU on child depressive symptoms. Possible explanations for these results are discussed along with the implications for clinicians and suggestions for future research.

**Trial registration:**

Clinical Trials identifier: NCT00635752

## Background

The role of parents has often been emphasized in models depicting factors associated with the development and maintenance of children’s reactions following traumatic experiences [1-3]. In line with this, several studies have shown the associations between parental reactions and their children’s symptom formation and adjustment after trauma [4-6]. More specifically, parental psychopathology is considered a risk factor for children’s development of posttraumatic stress disorder (PTSD) [7], and conversely, decreases in parental trauma-related symptoms has been found to predict lower levels of PTSD symptoms in children [8]. In addition, some treatment studies have investigated the association between parental symptoms and child outcomes [9,10]. For example, Weems and Scheeringa [9] found that the level of maternal depression pretreatment influenced child PTS-symptoms measured at follow-up in a sample of children aged 3 to 6 who were included either in a 12-weeks manualized CBT or a in a wait-list control group. Higher depression scores reported by the mothers were associated with increasing PTS-symptoms throughout the process. The results from this study may indicate that targeting parents’ depression may enhance treatment maintenance.

The critical role parents may have on children’s well-being is also reflected in the practice parameters for the treatment of children and adolescents with PTSD, where including parents as important agents of treatment change is recommended [11]. Adhering to this, parents are designated a significant role in Trauma-Focused Cognitive Behavioral Therapy (TF-CBT), a recommended treatment for children exposed to traumatizing events [12,13]. In TF-CBT, parents participate in both individual and con-joint sessions with the child [7]. One reason for involving parents in the treatment is to improve their parenting skills so they can be supportive and sensitive towards their child’s needs. Another reason is that, as parents may often experience strong negative emotions in relation to their child’s trauma, participation may alleviate parents’ own trauma specific reactions and depression [7].

Parents may react in several ways in relation to their child’s trauma. Feelings of distress, shame and guilt may be prominent [14]. They may also feel vulnerable without adequate coping skills to handle the situation and their child’s difficulties. Furthermore, they may feel depressed because of what has happened to their child [15]. Involving parents in their child’s treatment may provide them with hope that their child will fare well, in addition to reinforcing parental skills, thus possibly helping parents feel more competent and less helpless. Parents may also learn coping skills that they can use themselves to reduce stress and emotional reactions and alter maladaptive thoughts [7]. Alleviating stress may be especially helpful for parents who have experienced traumatizing events themselves or have been vicariously traumatized by their children’s experiences. Therefore, although TF-CBT is described as being primarily child-focused, the developers claim that involving parents in treatment may help them to cope better with their own difficulties as well [7,8].

TF-CBT studies examining the relationship between parents’ emotional reactions and child outcomes have shown mixed results. In an early study of sexually abused children, Cohen & Mannarino [16] found that there was a correlation between parental emotional reactions and child treatment outcome. The results did not differ between TF-CBT and non-directive supportive therapy, and the authors concluded that addressing parental distress related to their child’s trauma is important in providing effective treatment. In a later study, it was shown that parents of sexually abused children who participated in TF-CBT along with their children showed more improvements in their own levels of trauma-specific distress compared to parents of children receiving child-centered therapy (CCT), a non-directive child/ parent-centered treatment model [17]. Another study by Carrion, Kletter, Weems, Berry and Rettger [18] showed that when comparing a PTS treatment with a waitlist control group for youth exposed to interpersonal violence, caregivers’ anxiety and depression decreased in both conditions. In that study, however, there was only a significant effect of treatment on parental anxiety.

Furthermore, a study by Deblinger, Lippman & Steer [19] showed that including parents in TF-CBT was helpful for reducing child-reported depression and parent-reported behavior problems, but not in reducing child PTS symptoms. In line with this, King and colleagues [20] found that including parents in treatment did not improve the efficacy of TF-CBT on child PTS symptoms. The authors conclude that although trauma focused cognitive-behavioral treatment was useful for traumatized children; further research is required on the significance of caregiver involvement. In sum, these studies imply that parents seem to benefit themselves from engaging in their child’s treatment, but whether this mediates child outcomes is unclear.

Although TF-CBT is widely used and is the recommended treatment for children and youth exposed to traumas [12,13], few studies have actually looked into what change mechanisms that distinguish this method from other treatments. In particular, there is a lack of knowledge of what role parents may play in the treatment, whether parental emotional reactions and depression are significantly reduced during therapy and whether reductions in parental emotional stress and depression mediate the treatment difference between TF-CBT and TAU.

### Aims

The overarching goal of this study was to understand more about the role that parents play in treatment of traumatized children and youth by investigating the following issues: 1) whether caregivers reported changes in their own emotional reactions and depressive symptoms during therapy, and whether the reported changes differed between the two treatment conditions, and 2) whether the effect of treatment on child post-traumatic stress symptoms and child depressive symptoms was mediated by changes in parental emotional reactions and depressive symptoms. In line with previous studies, it was hypothesized that the level of parental depressive symptoms and emotional reactions would decline from pre- to post-therapy in both treatment conditions but that the reduction would be significantly larger in the TF-CBT group. Furthermore, it was expected that reductions in parental emotional and depressive reactions would mediate the effect of treatment on child PTS symptoms and child depressive symptoms.

## Method

The study builds upon a randomized effectiveness trial conducted in the period of April 2008 – December 2012 in which TF-CBT was shown to be more efficient in reducing child posttraumatic stress symptoms and depression than TAU [21]. Preliminary results from the same trial indicate that one mediating pathway of child PTS symptoms was changes in maladaptive appraisals. Eight child and adolescent mental health clinics were involved in the study. Four of the clinics were located in small cities, two in a large city and two in suburban areas. The results of the source trial showed that youth in the TF-CBT condition reported significantly lower levels of PTS symptoms (*d* = 0.51, *t* (154) = 3.30, *p* = .001), depressive symptoms (*d* = 0.54, *t* (154) = 2.79, *p* = .006) and general mental health symptoms (*d* = 0.45, *t* (152) = 2.46, *p* = .015) than participants receiving TAU [21].

### Procedures

The children and youth were referred to the eight community clinics according to regular practice (i.e., by their general practitioners or Child Protective Services). The inclusion criteria to the study were experiencing at least one potentially traumatizing event and suffering from PTS-symptoms above the cutoff score of 15 on the Child Post-Traumatic Symptom Scale (CPSS) [22]. The exclusion criteria were acute psychosis, active suicidal behavior, intellectual disability, or non-proficiency in the Norwegian language. The youth were screened for potentially traumatizing events and PTS symptoms at their respective clinics by a licensed psychologist who was blind to the treatment conditions. To assess participants’ trauma experiences, a short interview was developed using the questions from the Traumatic Events Screening Inventory for Children (TESI-C) [23]. The interview consists of 12 items that investigate the child’s exposure to different types of traumatic events. The psychologist coded ‘yes’ only if the child reported feeling scared, helpless, in despair or confused during or immediately after the event. Most of the children reported more than one traumatic experience, and were, therefore, asked to identify the trauma they experienced as being the worst. In addition, the youth had to report PTS symptoms above the cutoff score of 15 on the CPSS [22]. The time between trauma exposure and assessment needed to be at least four weeks. The parents accompanying the children were assessed for depressive symptoms and emotional reactions in response to the trauma their children had identified as worst. The parents completed the questionnaires primarily on a computer. If the parents did not participate in the particular sessions where the assessments were being scheduled, the questionnaire was sent home with the child or mailed to the caregiver, or the assessment was conducted over the telephone.

All assessments were performed at three time points: pre-treatment (T1), mid-treatment (after the 6th session; T2) and post-treatment (after the 15th session; T3). The therapies varied in lengths. On average, the T3-assessment was conducted 7.5 months after the T1-assessment, and the T2-assessment was conducted 3.5 months after the pre-assessment. Information about parental depression and/or/parental emotional reactions was collected from 130 (96.2%) of the parents at T1, 90 (66.6%) at T2 and 94 (69.6%) at T3. A few parents did not answer the questionnaires at T1 but answered the questionnaires at T2 and/ or T3. Thus, although only 130 parents were assessed at T1, the total number of parents assessed at one or more time points were 135. After receiving information about the study, both the children and parents provided written, active consent to participate. The study was approved by the Regional Committee for Medical and Health Research Ethics (REC). More details of study procedures are described in the source study [21].

### Participants

**Figure 1:**
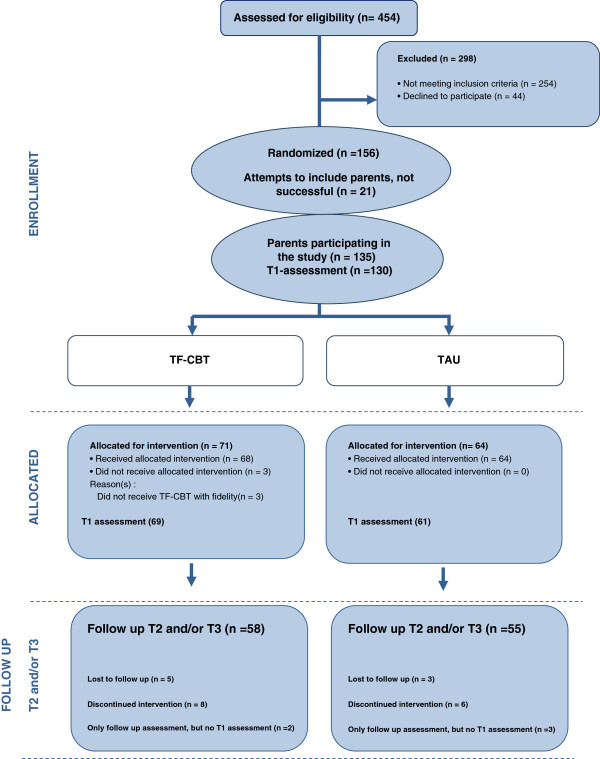
Flow chart of parents participating in the study.

A detailed description of the sample is presented in Table 1. The sample comprised 135 caregivers of 135 traumatized children and youth (see Figure 1). Most of the parents were mothers (*n* = 98, 72.6%); 22 (16.3%) were fathers and 15 (11.1%) were foster parents or other close relatives serving as caregivers. Most caregivers were Norwegian (*n* = 111, 82.2%); approximately one third (*n* = 46, 36.2%) had completed high school as their highest education level, and approximately half (*n* = 68, 54.4%) reported being employed full time.

**Table 1:** Description of participating parents and children

**Demographics of the parents (*N*** **= 135)**	***n *** **(%)**
**Person who completed the questionnaire (*n*** **= 135)**	
Mother	98 (72.6)
Father	22 (16.3)
Foster parents	12 (8.9)
Other	3 (2.2)
**Caregivers’ employment situations **^**a**^**(*n*** **= 125; lower *n*, due to missing data)**	
Working full time	68 (54.4)
Working part time	18 (14.4)
Job seeker	4 (3.2)
Student	4 (3.2)
Welfare recipient/Other	31 (24.8)
**Caregivers’ education**^**b **^**(*n*** **= 127; lower *n*, due to missing data)**	
Completed junior high school	17 (13.4)
Completed high school	46 (36.2)
Completed vocational school	15 (11.8)
<=4 years of college/university	41 (32.3)
> 4 years of college/university	8 (6.3)
**Caregivers’ ethnicity**	
Study country	111 (82.2)
Asian	11 (8.1)
Western European Countries	3 (2.2)
African Countries	4 (3)
South/ Central American Countries	2 (1.5)
Eastern European Countries	3 (2.2)
Northern American Countries	1 (0.7)
**Demographics of the children ( *N * = 135)**	***n *** **(%)**
**Child’s gender (*n*** **= 135)**	
Girls	108 (80)
Boys	27 (20)
**Child’s age (*n*** **= 135)**	
Range	10-18
Mean	*M* = 14.8 (*SD* = 2.2)
**Child’s living situation (*n*** **= 135)**	
Lives together with both parents	31 (23)
Lives equally with mother and father, but parents are divorced	4 (3)
Live most or only with mother	70 (51.9)
Live most or only with father	13 (9.6)
Foster care	12 (8.9)
Other (alone, institution, with boyfriend)	5 (3.7)
**Household income (*n*** **= 117; lower *n*, due to missing data)**	
< NOK 200,000 (< USD 35,000)	17 (14.5)
[NOK 200,000, NOK 500.000) ([USD 35,000, 87,000)]	46 (39.3)
[NOK 500,000,1.000.000] ([USD 87,000, 174,000)]	39 (33.3)
> NOK 1,000,000 (>USD 174,000)	9 (7.7)
Do not know	6 (5.1)
**Trauma groups, Child’s primary (worst) trauma (*n*** **= 135)**	
Accident	3 (2.2)
Sudden death/ injury of a close person	25 (18.5)
Hospitalization	1 (0.7)
Extrafamilial violence	23 (17)
Robbed	1 (0.7)
Witness physical abuse inside family	5 (3.7)
Exposed to physical abuse inside family	38 (28.1)
Sexual abuse outside family	28 (20.7)
Sexual abuse inside family	11 (8.1)
**Months since worst trauma occurred (*n*** **= 135)**	
Range	1-138
Mean	*M* = 30.0. (*SD* = 32.8)
**Child’s total number of traumatic experiences *(n*** **= 135)**	
Range	1-8
Mean	*M* = 3.5 (*SD* = 1.7)
**Child’s scores on the CAPS-CA, T1 (*n*** **= 135)**	
Range	*9-125*
Mean	*M* = 60.4 (*SD* = 20.3)
**Child’s scores on the CPSS, T1 (*n*** **= 135)**	
Range	15-46
Mean (SD)	*M* = 29.9 (*SD* = 7.6)

^*a*^In 2012, 68% of the (country) population worked full-time.

^*b*^In 2010, the highest level of education for 30% of the (country) population was completing high school.

The children ranged in age from 10 to 18 years (*M* age = 14.8, *SD* = 2.2), and 108 (80.0%) were girls. More than half of the children lived in single-parent households headed by their mothers (*n* = 70, 51.9%). All of the youth had experienced at least one traumatic event that occurred ≥ four weeks before the study inclusion and had developed significant PTS symptoms assessed using the Child Post-Traumatic Symptom Scale (CPSS). On average, the participants reported having been exposed to 3.5 (*SD* = 1.7, range 1–8) different types of traumatic events. When asked to identify their worst trauma, 43 (31.8%) reported being exposed to domestic violence, 23 (17%) had experienced extra-familial violence, 28 (20.7%) sexual abuse outside the family, 11 (8.1%) had been exposed to sexual abuse within the family, 25 (18.5%) had experienced traumatic loss (i.e. sudden death or severe illness of a close person), and the remaining 5 participants (3.6%) had been exposed to accidents or other forms of non-interpersonal traumas.

### Treatment conditions

A computer-generated randomized block procedure at each clinic was used to randomly assign the participants to either TF-CBT or TAU. The TF-CBT therapists (*n* = 26) volunteered to receive training in TF-CBT and to provide therapy to the participants who were randomly selected to receive TF-CBT. The TAU therapists (*n* = 45) provided their usual treatment. All therapy sessions were audio recorded to enable treatment fidelity coding. Trained TF-CBT therapists coded fidelity by using the TF-CBT Fidelity Checklist developed by the treatment developers [24]. In this checklist, 11 items are rated as either “present” or “absent”. These items follow the treatment components of TF-CBT. The core components (psychoeducation, relaxation skills, affect regulation, instruction in the cognitive triangle, working through the trauma narrative, working with dysfunctional thoughts, and the parenting component) had to be completed in order for a therapy to be defined as TF-CBT. In cases where there was any uncertainty or questions about the fidelity, this was determined by consensus. Based on these criteria, three TF-CBT cases failed to reach the level of required fidelity. In the TF-CBT group, all sessions in all cases were coded for fidelity. The same Fidelity Checklist was used for the TAU-cases where 392 sessions were coded. The main aim by reviewing the TAU-cases was to ensure that the therapists were not providing TF-CBT. At least five sessions (the first, second, third, sixth, and ninth sessions) were coded in each TAU case. Additional sessions were investigated if elements of the core components were provided also in the TAU-sessions. Although some TAU cases used certain elements similar to the TF-CBT-components, none of the TAU cases met the adherence criteria for TF-CBT.

### TF-CBT

TF-CBT is a 12–15 session, trauma-specific treatment consisting of psycho-education, learning relaxation skills, affective modulation skills, cognitive coping skills, working through the trauma narrative, cognitive processing, in vivo mastery of trauma reminders, and enhancing safety and future developments, coupled with a parental component. The parental component is focused on improving parenting skills; each treatment component provided to the child is also demonstrated for the parent in both parallel and con-joint sessions [7].

The TF-CBT therapists consisted of 21 (80.8%) psychologists, two (7.7%) psychiatrists, two (7.7%) educational therapists and one (3.8%) social worker. The therapists had 10.2 years of experience on average (*SD* = 6.4 years, range 3–28 years). They were all trained in the treatment protocol by the treatment developers and other approved TF-CBT trainers. The TF-CBT therapists each treated an average of 3.0 (*SD* = 1.4, range 1–6) of parent–child dyads. All therapists received four to six days of training, read the treatment manual [7] and completed a web-based course on trauma-focused cognitive behavioral therapy (http://www.musc.edu/tfcbt, 2013).

Of the 61 completed TF-CBT cases, caregivers participated in 56 cases (91.8%). In the five cases in which parents were not involved in the therapy, the children were older than 16 years. In these cases, the parents were perpetrators, had substance abuse problems, were struggling with their own mental health problems, and/or the youth lived alone without parental contact. When drop-outs were included, the parents participated in 60 of 71 cases (84.5%).

### TAU

The TAU therapists provided the treatment they considered most suitable in each individual case. In total, 45 TAU therapists volunteered to participate, and each therapist treated an average of 1.7 (*SD* = 1.3, range 1–9) participants (either individual youth or parent–child dyads). They described their theoretical orientations as psychodynamic (*n* = 17, 45.9%), cognitive-behavioral (*n* = 11, 29.7%), and family/systemic (*n* = 9, 24.3%). There were 23 (51.1%) psychologists, 12 (26.7%) social workers, eight (17.8%) educational therapists, and two (4.4%) psychiatrists. In 35 (*n* = 67.3%) of the 52 completed TAU cases, parents were involved in more than three sessions. In nine of these cases (25.7%), the parents attended the sessions together with the children; five (14.3%) had sessions alone with their child’s therapist, and 21 (60%) had some combination of the above. When including the drop-outs in these calculations, parents participated in 39 of 64 (60.9%) initiated TAU therapies. Of these 39 therapies, 10 (25.6%) parents attended the sessions together with the children, six (15.4%) had sessions alone with their child’s therapist, and 23 (58.9%) had some combination of the above.

### Parent measures

#### Parent emotional reaction questionnaire (PERQ)

The PERQ measures parents’ emotional reactions to their children’s traumatic experiences [25]. The parent rates a specific emotional reaction on a 5-point Likert scale ranging from never to always (e.g., 1 = never, 5 = always), depending on how often they have experienced the reaction during the last two weeks. The original instrument consisted of 15 items. However, the last item in the scale, “I feel guilty that I did not know about the trauma sooner,” was excluded because most of the parents in this study learned about the trauma immediately after it occurred. The scale’s authors have previously found the PERQ to have good validity and reliability. Internal consistency for the scale was calculated to be .87, and test-retest reliability was .90 [25]. The instrument has been used in several treatment studies [16,26-28].

#### Center for epidemiologic studies depression scale (CES-D)

The CES-D is a 20-question self-reporting instrument designed to measure depressive symptoms in the general adult population [24]. Parents are instructed to report how often they have experienced each of 20 depressive symptoms during the last week on a 4-point Likert scale ranging from 0–3 (e.g., 0 = rarely or none of the time, 3 = most or all of the time). Scores of 16 or above are considered indicative of clinically significant symptoms of depression [29]. The scale has also been found to have adequate concurrent validity and split-half and coefficient alpha reliability for both general populations and clinical samples [24]. The current study yielded an internal consistency score of α = .91.

### Child measures

#### The clinician-administered PTSD scale for children and adolescents (CAPS-CA)

The CAPS-CA is a structured clinical interview for children and adolescents; it assesses the frequency and intensity of the 17 DSM-IV-defined PTSD symptoms [30,31]. Items are scored on 5-point frequency scales (e.g., from 0 = “None of the Time” to 4 = “Most of the Time”) and 5-point intensity rating scales (e.g., from 0 = “Not a Problem” to 4 = “A Big Problem, I Have to Stop What I Am Doing”) for the past month. Items are scored based on both the youth’s answers and on the clinician’s judgment. The total scale showed satisfactory internal consistency (α = .90).

#### Mood and feelings questionnaire (MFQ)

MFQ is a 34-question self-report questionnaire designed to assess depressive symptoms in children and youth between eight and 18 years of age [32]. The questionnaire measures the full range of DSM IV diagnostic criteria for depressive disorders as well as additional items reflecting common affective, cognitive, and somatic features of childhood depression. The child rates the problem frequency during the last two weeks using a three-point scale from 0–2 (0 = Not true, 1 = Sometimes true, 2 = True). In this sample the instrument showed good internal consistency (α = .91).

### Data analyses

Descriptive statistics were applied to investigate the sample characteristics. Effect sizes, using Cohen’s *d* (*d*), were calculated to show the strength and magnitude of change in parental emotional reactions (measured by PERQ) and in parental depressive scores (measured by CES-D) within each treatment group, as well as the difference between the interventions. Mixed effects models were estimated to investigate change in the different parental scores across time. Mixed effects models handle missing data under the missing at random (MAR) assumption [33]. The approach takes into account the nested nature of the data and has the advantage of estimating a measure of random variation both between and within the participants [34]. The models analyzed two parental dependent variables of parental emotional reactions and parental depressive symptoms in separate analyses, and the independent variables were therapy condition and time, including a condition by time interaction. Within the mixed effects models, intention-to-treat (ITT) analyses were conducted, meaning that all recruited parents (*n* = 135, including drop-outs and the few TF-CBT cases failing to reach the acceptable level of fidelity) were analyzed in the condition into which they were originally randomized.

**Figure 2:**
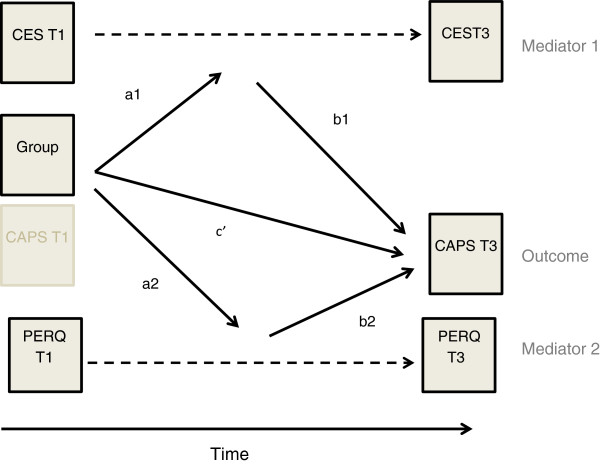
Example of the mediation model; parental mediation on child PTS-symptoms.

Multiple mediation models, which were devised by Preacher and Hayes [35], were used to examine the mediating role of change in parental emotional reactions and parental depressive symptoms in the effectiveness of TF-CBT on TAU. The two mediators in the models were; 1) the change in parental emotional reactions scores 2) the change in parental depressive scores. The mediation models were estimated two times with different outcome measures: 1) child PTS symptoms at T3 and 2) child depressive symptoms at T3 (see Figure 2 for example of the mediation model on child PTS symptoms).

The bootstrap resampling method was applied using 10,000 re-samples of the data [36], and bootstrap percentile confidence intervals were computed and relationships were considered as significant if 0 was outside these intervals. The mediation analysis comprised two different models: one model for the mediator, which included the a-path that indicated the relationship between the main independent variable (IV) and the mediator (M), and one model for the outcome, including the b-path showing the relationship between the M and the dependent variable and the c’-path showing the relationship between the IV and DV, while controlling for the M [35,36]. The main reason for applying the mediation model was not to look into the different paths separately but to investigate the indirect effect of change in parental emotional reactions and depression on child outcomes. As such, a significant indirect effect could be present even though the relationships represented in the individual paths were not significant. The mediator analyses were conducted only on the completed therapy cases. The treatment of missing data in the mediation analyses, provided by Mplus was full information maximum likelihood (FIML) under the missing at random (MAR) assumption [37].

We computed the intra-class correlation (ICC) within the data set because more than one dyad of parent and child had the same therapist, and because more than one dyad of child and parent was treated at the same clinic. In general, a high ICC requires the application of multilevel modeling (HLM) because this indicates that much variation in the outcome variable is due to nesting groups. A need to consider using HLM is present if ICC is 0.25 or above [38,39]. All ICCs for the therapist and clinical levels in child outcomes and the mediators were below .05, which is well below the recommended level of. 25 [38], therefore clustering of therapist and clinic was not taken into account in the analyses. Mixed effects models used the R (The R Foundation for Statistical Computing, Vienna, Austria) package nlme, mediation analyses used Mplus [37], while SPSS, version 17 (IBM SPSS Statistics, 2011) was used for other analyses.

## Results

### Attrition and baseline comparisons

Of the 135 parents and children dyads included in the study, 22 (16.3%) dropped out of therapy before session six. The drop-out rate was not significantly different in the two treatment conditions (*p* = .464). There were no significant differences between the retention group and the attrition group regarding basic characteristics, such as children’s gender (*p* = .816) and age (*p* = .136), parental background information (parents’ ethnicity; *p* = .914 parents’ education; *p* = .439 and parents’ employment situation; *p* = .652), the child’s total number (*p* = .896) and type (*p* = 925) of experienced traumas, or any outcome variables for the children (CAPS; *p* = .982 and MFQ; *p* = .111) at baseline. The parents in the retention group and attrition group did not differ significantly from one another on the parental outcome measures either (PERQ; *p* = .181 and CES-D; *p* = .914).

### Comparisons of therapists in TF-CBT and TAU

There was a statistically significant difference between the groups in terms of therapists’ years of experience in which therapists in the TAU group reported significantly more years of experience (*M* = 15.87, *SD* = 12.89) than did the therapists in the TF-CBT group (*M* = 9.69, *SD* = 5.73), *p* < .001. Furthermore, there were significant differences in therapists’ educational background as there were more psychologists in the TF-CBT condition (*p* < .001), and the TF-CBT therapists had significantly more participant cases compared to TAU (*p* < .001).

### Change analyses

Means and standard deviations divided into treatment condition and time are presented in Table 2, and treatment effects and interaction effects are presented in Table 3. There was a main effect of time in both treatment groups on parental depressive scores, which indicated that parents had significant reductions in their depressive scores both in TF-CBT, *t* (171) = −5.40, *p* < .001, and in TAU: *t* (171) = −2.14, *p* = .034. There was no significant main effect of treatment condition at the end of treatment, indicating that parents in the two groups did not differ significantly from one another regarding their depressive scores at the end of treatment; *t* (132) = 1.69, *p* = .094. The interaction between time and group, however, was significant, indicating that the slopes of the different conditions over time were significantly different from each other in the two conditions with a superior effect of TF-CBT at T2 and T3 (*p* = .022).

**Table 2:** Descriptions of parental outcome variables: means and SD by treatment condition and time and effect size

	***Therapy as usual***				***TF-CBT***				
***Outcome***	***Time 1***	***Time 2***	***Time 3***	***d***_***1***_	***Time 1***	***Time 2***	***Time 3***	***d***_***2***_	***d***_***3***_
	***M (SD)***	***M (SD)***	***M (SD)***		***M (SD)***	***M (SD)***	***M (SD)***		
***CES-D***	17.25 (9.75)	17.60 (12.52)	13.39 (11.91)	0.40	*17.55 (12.28)*	13.26 (10.98)	10.96 (10.35)	0.54	0.22
	*n* = 61	*n* = 43	*n* = 44		*n* = 66	*n* = 47	*n* = 48		
***Perq***	*35.22 (11.09)*	*31.60 (11.37)*	*31.64 (11.39)*	0.32	*37.00 (9.97)*	*31.16 (10.02)*	*28.33 (10.28)*	0.87	0.31
	*n = 58*	*n = 43*	*n = 45*		*n = 69*	*n = 45*	*n = 48*		

*Note*. PERQ = Parental Emotional Reaction Questionnaire, CES-D = Center for Epidemiologic Studies.

Depression Scale.

*d*_*1*_ = calculated based on differences between T1 and T3 in the TAU-condition: $\frac{\mathrm{TAU}\;T1-\mathrm{TAU}\;T3}{\mathrm{SD}\;\mathrm{TAU}\;T1}$.

*d*_*2*_ = calculated based on differences between T1 and T3 in the TF-CBT-condition: $\frac{\mathrm{TFCBT}\;T1-\mathrm{TFCBT}\;T3}{\mathrm{SD}\;\mathrm{TFCBT}\;T1}$.

*d*_*3*_ = calculated based on differences between the two conditions at $T3:\frac{\mathrm{TAU}\;T3-\mathrm{TFCBT}\;T3}{\mathrm{SD}\;\mathrm{pooled}}$ where $\mathrm{SD}\;\mathrm{pooled}=\sqrt{\frac{\left(n_{1}-1\right)SD_{1}^{2}+\left(n_{2}-1\right)SD_{2}^{2}}{n_{1}+n_{2}-2}}$.

**Table 3:** Treatment effects a) between times within each treatment condition and b) between treatments conditions

	***Treatment effect:***			***a) Within group analyses***		
	***TF-CBT***			***TAU***		
***Outcome***	***Estimate***	***95% CI***	***p***	***Estimate***	***95% CI***	***p***
***CES-D***	-3.88	- 6.37 to -1.38	.003	0.68	-1.90 to 3.28	.603
*T2 vs T1*	-6.73	- 9.19 to -4.27	<.001	-2.78	-5.35 to -0.21	.034
*T3 vs T1*						
***PERQ***						
*T2 vs T1*	-5.80	- 8.51 to -3.01	<.001	-3.83	-6.71 to -0.95	.010
*T3 vs T1*	-8.71	-11.35 to -6.02	<.001	-4.27	-7.06 to -1.49	.003
	***Treatment Effect***		***b Between group***		***Interaction: Time by Group***	
***Outcome***	***Estimate***		***95% CI***	***p***	***p***	
***CES-D***						
*TF-CBT vs TAU T3*	3.62		-0.61 to 7.86	.094	.022	
***PERQ***						
*TF-CBT vs TAU T3*	2.95		-1.12 to 7.01	.154	.078	

*Note*. PERQ = Parental Emotional Reaction Questionnaire, CES-D = Center for Epidemiologic Studies Depression Scale.

There was a main effect of time in both treatment groups for PERQ scores, indicating that parents had a significant reduction in their own distress reactions from pre- to post-therapy in TF-CBT: *t* (167) = −6.50, *p* < .001, as well as in TAU; *t* (167) = −3.03, *p* = .003. However, even though the TF-CBT parents reported lower levels of distress at the end of therapy, this difference was not statistically significant; *t* (74) = 1.43, *p* = .154. There was no significant time by group interaction either (*p* = .078).

### Mediation analyses

The first model, using the children’s PTS symptoms (CAPS-CA) as an outcome variable, did not reveal a significant indirect effect of treatment via the mediators together (CES-D and PERQ): estimate = 1.08, 95% bootstrap percentile CI [−1.59, 6.29]. Examining the depressive symptoms (CES-D) and the emotional reactions (PERQ) separately showed that neither of the scores on the individual scale revealed any significant results. CES-D: estimate = 2.27, 95% bootstrap percentile CI [−0.40, 9.55], and PERQ: estimate = −1.19, 95% bootstrap percentile CI [−6.85, 0.72].

The second mediation model was applied using the child depressive scores (MFQ) as the outcome. A significant indirect treatment effect was found using the two mediators of change in child depression (CES-D) and parental emotional reactions (PERQ) together: estimate = 2.03, 95% bootstrap percentile CI [0.11, 4.97], but only one of the mediators had a significant individual mediating effect: CES-D; estimate; 2.86, 95% bias corrected CI [0.57, 6.76]. No significant individual mediating effect of PERQ was found; estimate; −0.82, 95% bootstrap percentile CI [−3.55, 0.27]. Furthermore, worth mentioning was that there was a significant relationship between overall change in parental depressive symptoms and child depressive symptoms; estimate; 0.61, bias corrected CI [0.23, 0.93] (cf. the b-path in the model). The results from the mediation results are presented in Table 4 and Table 5.

**Table 4:** Parental mediation on child PTS (with Bootstrap Method)

**Effect**	**Estimate**	**95% CI Bootstrap percentile**
***a***	-	-
CES-D	3.83	−0.05 to 7.81
PERQ	3.35	−1.01 to 7.67
***b***	-	-
CES-D	0.59	−0.24 to 1.41
PERQ	−0.36	−1.06 to 0.38
***c***’	10.33	0.14 to 20.60
***Total Indirect***	1.08	−1.59 to 6.29
CES-D	2.27	−0.40 to 9.55
PERQ	−1.19	−6.85 to 0.72

Note. CES-D = Center for Epidemiologic Studies Depression Scale, PERQ = Total scale of Parental Emotional Reaction Questionnaire.

*a* = the relationship between the IV and the M, *b* = the relationship between the M and the DV, *c’* = the relationship between the IV and DV, while controlling for the M, Total indirect: The indirect effect of the M on the relationship between IV and D.

IV = Group.

**Table 5:** Parental mediation on child depression (with Bootstrap Method)

**Effect**	**Estimate**	**95% CI Bootstrap percentile**
***a***	-	-
CES-D	4.67	0.94 to 8.61
PERQ	2.88	−1.46 to 7.24
***b***	-	-
CES-D	0.61	0.23 to 0.93
PERQ	−0.29	−0.66 to 0.10
***c***’	6.19	−0.59 to 11.85
***Total Indirect***	2.03	0.11 to 4.97
CES-D	2.86	0.57 to 6.76
PERQ	−0.82	−3.55 to 0.27

Note. CES-D = Center for Epidemiologic Studies Depression Scale, PERQ = Total scale of Parental Emotional Reaction Questionnaire.

*a* = the relationship between the IV and the M, *b* = the relationship between the M and the DV, *c’* = the relationship between the IV and DV, while controlling for the M, Total indirect: the indirect effect of the M on the relationship between IV and D.

IV = Group.

## Discussion

The primary aim of this study was to improve our understanding of the role that a parent’s own distress and depressive reactions plays in the treatment of traumatized children and youth. Specifically, we wanted to investigate 1) whether caregivers reported changes in their own emotional reactions and depressive symptoms during the therapy process and whether the changes differed between TF-CBT and TAU and 2) whether the effect of treatment on child PTS symptoms and child depressive symptoms was mediated by change in parental emotional reactions and depressive symptoms. The results showed that parents in both conditions experienced a significant reduction in emotional reactions as well as in depressive reactions from pre- to post-therapy. The investigation of change in parental emotional reactions and depression as possible mediators of the treatment effect showed that the reactions did significantly mediate the child depressive symptoms, but not the child PTS reactions.

The fact that parents in both treatment groups experienced an alleviation of their own emotional reactions and depression was as expected and in line with previous studies. The alteration in parental reactions in both groups may be attributed to enhanced feelings of hope and expectations that professional support will help their children function and cope better in the future. Because treatment expectancies have been shown to relate to outcomes in adult treatment studies [40-42], it may be that positive expectancies regarding their children’s treatment outcomes could indirectly result in less distress and fewer depressive reactions in parents as well. It may also be that having another person participate in and share the responsibility for their children’s well-being, may evoke relief within parents and help them feel less vulnerable and alone. One could also expect that the reduction in parental symptoms was a result, at least partly, of the children’s improvement. However, because only one single association between parental and child improvement was found in this sample (parental depression did relate to child depression), this explanation was not supported.

Contrary to our expectations, change in parental emotional reactions and depression did not seem to mediate the effect of treatment on children’s post-traumatic stress symptoms. However, the reactions mediated the child’s depressive symptoms significantly. We are unaware of any other studies that have examined parental reactions as a mediator of childhood trauma treatment outcome. However, the findings may be seen in light of the studies by King et al. [20] and Deblinger et al. [19] that found that caregiver participation in therapy did not have any additional effect on children’s PTS symptoms. Deblinger et al. [19] investigated the various effects of mother and child participation in CBT for sexually abused children. Three different treatment conditions were evaluated: 1) mother alone 2) mother and child together and 3) child alone. The study showed that the greatest reduction in PTS symptoms occurred when the child was present in the therapeutic process, and that the caregiver’s participation did not influence the child’s PTS improvement [19]. However, parental involvement did have an additional effect on the children’s depressive symptoms and children’s externalizing behavior. It may be that treating child depression and child externalizing problems involves different change mechanisms than does treating post-trauma symptoms and other anxiety disorders. In fact, La Greca, Silverman, and Lochman [43] and Silverman and colleagues [13] point out that there is little evidence within the child anxiety literature that targeting parental skills alone and involving parents in treatment contributes to positive child outcomes. This may also be the case for children who suffer from PTS reactions after trauma.

Targeting maladaptive appraisals, on the other hand, has been found to mediate PTS symptoms in samples of traumatized children and youth [44]. This is also in line with cognitive theories on PTSD sequelae claiming that maladaptive appraisals and trauma memory processing characteristics contribute to developing and maintaining posttraumatic stress reactions [45]. In addition, preliminary results from the current effectiveness trial indicate that changes in maladaptive appraisals mediate the treatment effect on child PTS symptoms. Thus, although it may seem surprising from a developmental perspective that changes in parental emotional reactions were not found to be significantly related to the treatment effect on child PTS symptoms, the findings of the current study may indicate that other factors may be more important in mediating the effect of treatment on child PTS-symptoms. Still, the parents’ alleviation did play a role in explaining why the youth were less depressed in TF-CBT than in TAU.

### Limitations and future research

Some limitations in this study are worth noting. First of all, although it is an advantage that participants were measured at more than two time points, associations might have appeared clearer within a wider timeframe. Transactions of reactions between parents and their children are complex, and with more frequent measurements and different and multiple measures, e.g., other than self-reported measures, it would have been possible to capture a more complete picture of the interplay between the development of parental stress and children’s trauma-related symptoms over time. Future studies should aim to investigate longitudinal trajectories of parental and child’s reactions with multiple measures over time.

Second, although the age range was wide (10–18 years), the majority of participants were adolescents, and the mean age was 14.8 years. It would be interesting to see whether the present results apply to younger samples as well, especially because the potential influence of parents’ emotional reactions on child development may differ between developmental phases. Third, the study comprised a sample of traumatized children and youth commonly seen in community mental health clinics. Still, the exclusion of participants who did not speak Norwegian could limit the generalizability of these findings to other ethnic groups. In addition, though the mixture of different traumatic experiences in this sample mirrors the population commonly seen in community mental health clinics and is considered a strength of the study, this heterogeneity could also interfere with our findings. Furthermore, although the dropout rates did not differ significantly between the two treatment conditions, the participants’ decision to terminate treatment could have influenced the results. A small sample size with a high dropout rate also deserves to be mentioned as study limitations. Concerning the negative findings, investigation of confidence intervals is essential in order to evaluate whether the findings are conclusively or inconclusively negative. The CIs displayed within these results were relatively small, indicating that they represent conclusive negative findings. However, this observation cannot be concluded with certainty. Lastly, the therapists, especially in the TAU-condition, differed in their theoretical orientations and educational qualifications. In addition, most therapists (in both conditions) treated more than one case. One can neither assume parental ratings nor child’s ratings to be fully independent of this. Preferably, therapists should have been randomized into one of the two conditions to minimize therapist effects. A focus and examination of whether and how therapist factors influence the treatment results need more attention in future treatment research.

Overall, this study’s findings suggest the need for future research on the mechanisms of change in treatments for traumatized children and youth. Such research could inform theoretical approaches and aid clinicians in their work. It would also be of interest to further explore whether emotional reactions differ between mothers and fathers and between sons and daughters and to determine how parents’ own trauma history may influence their reactions and parenting behaviors. Future studies should also seek to understand why parents may experience less emotional stress and depressive reactions when their child receives therapy. A qualitative approach to explore how parents experience the therapy provided to their children would be a valuable contribution in this respect. The impact of time is also a potential area for future research.

## Conclusion

The findings show that parents also reap benefits when their children receive treatment. However, although parents’ emotional reactions and depressive symptoms were reduced during the therapy, this did not seem to be related to the difference between TF-CBT and TAU on child PTS-symptoms. However, the parental alleviation did mediate child depressive symptoms significantly.

Even though changes in parental reactions were not significantly related to treatment outcome on child PTS symptoms, it is important to emphasize that it may still be helpful to include parents in therapy with traumatized youth. Clinicians should be aware of, and open to, the fact that traumatized children and youth may behave in manners that are difficult for parents to handle. Because the children’s behavior may differ from the norm, parents may need counseling on how to respond to these changes and on how to enhance their parenting skills. They may also need help in how to cope with their own responses to the child’s trauma experiences because excessive feelings of distress, guilt, shame or sadness may contribute to the maintenance of child post-trauma reactions. On the other hand, although children may be negatively influenced by excessive parental emotional strain, such reactions can also be a sign of parental sensitivity and concern. Helping parents to find a good balance between caring and overreacting may be an important task for clinicians.

For clinicians treating traumatized youth suffering from PTSD, the results of the study may support the importance of working individually with children to reduce trauma-related symptoms. The results also indicate that involving parents in treatment may help parents to reduce their own emotional reactions.

## Abbreviations

TF-CBT: Trauma-focused cognitive behavioral therapy; TAU: Therapy as usual; PTS symptoms: Post-traumatic stress symptoms; PERQ: Parental emotional reaction questionnaire; CES-D: Center for epidemiologic studies depression scale; CAPS-CA: Clinician-administered ptsd scale for children and adolescents; MFQ: Mood and feelings questionnaire.

## Competing interests

The authors declare that they have no competing interests.

## Authors’ contributions

TH contributed to collecting data, performing the statistical analyses and drafted the manuscript. TKJ designed and coordinated the study and contributed to the manuscript. TW-L contributed to the statistical analyses and contributed with critical comments on the manuscript. All authors read and approved the final manuscript.

## References

[B1] La Greca AM Silverman WK Vernberg EM Prinstein MJ Symptoms of posttraumatic stress in children after Hurricane Andrew: a prospective study J Consult Clin Psychol 1996 64 712 723 8803361 10.1037//0022-006x.64.4.712

[B2] Pynoos RS Steinberg AM Wraith R Cicchetti D, Cohen DJ A developmental model of childhood traumatic stress Developmental Psychopathology, Volume 2 1995 Oxford, England: John Wiley & Sons 72 95

[B3] Scheeringa MS Zenah CH A relational perspective on PTSD in early childhood J Trauma Stress 2001 14 799 815 10.1023/A:1013002507972 11776426

[B4] Dyb G Jensen TK Nygaard E Children’s and parents’ posttraumatic stress reactions after the 2004 tsunami Clin Child Psychol Psychiatry 2011 16 1 14 10.1177/1359104510391048 21565871

[B5] Morris A Delahanty D The Association Between Parent PTSD/Depression Symptoms and Child PTSD Symptoms: A Meta-Analysis J Pediatr Psychol 2012 37 1076 1088 10.1093/jpepsy/jss091 23019132

[B6] Trickey D Siddaway AP Meiser-Stedman R Serpell L Field AP A meta-analysis of risk factors for post-traumatic stress disorder in children and adolescents Clin Psychol Rev 2012 32 122 138 10.1016/j.cpr.2011.12.001 22245560

[B7] Cohen JA Mannarino AP Deblinger E Treating Trauma and Traumatic Grief in Children and Adolescents 2006 New York: Guilford Publications

[B8] Pine DS Cohen JA Trauma in children and adolescents: risk and treatment of psychiatric sequelae Biol Psychiatry 2002 51 519 531 10.1016/S0006-3223(01)01352-X 11950454

[B9] Silverman WK Kurtines WM Ginsburg GS Weems CF Lumpkin PW Carmichel DH Treating anxiety disorders in children with group cognitive-behavioral therapy: a randomized clinical trial J Consult Clin Psychol 1999 67 995 1003 10596522 10.1037//0022-006x.67.6.995

[B10] Weems CF Scheeringa MS Maternal depression and treatment gains following a cognitive behavioral intervention for posttraumatic stress in preschool children J Anxiety Disord 2013 27 140 146 10.1016/j.janxdis.2012.11.003 23376601 PMC3578069

[B11] Cohen JA Bukstein O Walter H Benson RS Chrisman A Farchione TR Hamilton J Keable H Kinlan J Schoettle U Siegal M Medicus J Stock S Medicus J Practice parameter for the assessment and treatment of children and adolescents with posttraumatic stress disorder J Am Acad Child Adolesc Psychiatry 2010 49 414 430 20410735

[B12] NICE NICE clinical guideline on post-traumatic stress disorder 2005 http://www.nice.org.uk/Guidance/CG26

[B13] Silverman WK Ortiz CD Viswesvaran C Burns BJ Kolko D Putman FW Amaya-Jackson L Evidence-based psychosocial treatments for children and adolescents exposed to traumatic events J Clin Child Adolesc Psychol 2008 37 156 183 10.1080/15374410701818293 18444057

[B14] Holt T Cohen J Mannarino A Parental emotional response to children’s traumas J Aggress Maltreat Traumain press

[B15] Elliot AN Carnes CN Reactions of nonoffending parents to the sexual abuse of their child: a review of the literature Child Maltreat 2001 6 314 331 10.1177/1077559501006004005 11675815

[B16] Cohen JA Mannarino AP Factors that mediate treatment outcome of sexually abused preschool children J Am Acad Child Adolesc Psychiatry 1996 35 1402 1410 10.1097/00004583-199610000-00028 8885595

[B17] Cohen JA Deblinger E Mannarino AP Steer RA A multisite randomized controlled trial for children with sexual abuse related PTSD symptoms J Am Acad Child Adolesc Psychiatry 2004 43 393 402 10.1097/00004583-200404000-00005 15187799 PMC1201422

[B18] Carrión VG Kletter H Weems CF Berry RR Rettger JP Cue-centered treatment for youth exposed to interpersonal violence: A randomized controlled trial J Trauma Stress 2013 26 654 662 10.1002/jts.21870 24490236

[B19] Deblinger E Lippman J Steer R Sexually abused children suffering posttraumatic stress symptoms: Initial treatment outcome findings Child Maltreat 1996 1 310 321 10.1177/1077559596001004003

[B20] King NJ Tonge BJ Mullen P Myerson N Heyne D Rollings S Martin R Ollendick T Treating sexually abused children with posttraumatic stress symptoms: a randomized clinical trial J Am Acad Child Adolesc Psychiatry 2000 39 1347 1355 10.1097/00004583-200011000-00008 11068889

[B21] Jensen TK Holt T Ormhaug SM Egeland K Granly L Hoaas L Hukkelberg SS Indregaard T Stormyren S Wentzel-Larsen T A randomized effectiveness study comparing trauma-focused cognitive behavioral therapy with therapy as usual for youth J Clin Child Adolesc Psychol 2013 0 0 1 14 10.1080/15374416.2013.822307 PMC4037845 23931093

[B22] Foa EB Johnson KM Feeny NC Treadwell KR The Child PTSD Symptom Scale:a preliminary examination of its psychometric properties J Clin Child Psychol 2001 30 376 384 10.1207/S15374424JCCP3003_9 11501254

[B23] Ribbe D Stamm BH Psychometric review of Traumatic Event Screening Instrument for Children (TESI-C) Measurement of Stress, Trauma, And Adaptation 1996 Lutherville, MD: Sidran Press 386 387

[B24] Deblinger E Cohen JA Mannarino AP Murray LA Epstein C TF-CBT Fidelity Checklist 2008 Stratford, NJ: UMDNJ-SOM CARES Institute Unpublished instrument

[B25] Mannarino AP Cohen JA Family-related variables and psychological symptom formation in sexually abused girls J Child Sex Abus 1996 5 105 120 10.1300/J070v05n01_06

[B26] Cohen JA Mannarino AP Predictors of treatment outcome in sexually abused children Child Abuse Negl 2000 24 983 994 10.1016/S0145-2134(00)00153-8 10905421

[B27] Cohen JA Mannarino AP Knudsen K Treating childhood traumatic grief: a pilot study J Am Acad Child Adolesc Psychiatry 2004 43 1225 1233 10.1097/01.chi.0000135620.15522.38 15381889

[B28] Deblinger E Mannarino AP Cohen JA Steer RA A Follow-up study of a mutisite, randomized, controlled trial for children with sexual abuse-related PTSD Symptoms J Am Acad Child Adolesc Psychiatry 2006 45 1474 1484 10.1097/01.chi.0000240839.56114.bb 17135993

[B29] Radloff LS The CES-D Scale: a self report depression scale for research in the general population Appl Psych Meas 1977 1 384 401

[B30] Nader K Kriegler JA Blake DD Pynoos RS Newman E Weathers F Clinician Administered Ptsd Scale, Child And Adolescent Version 1996 White River Junction, VT: National Center for PTSD

[B31] Nader K Newman E Weathers F Kaloupek DG Kriegler J Blake D Clinician-Administered PTSD Scale for Children and Adolescents (CAPS-CA) 2004 Los Angeles, CA: Western Psychological Press

[B32] Angold A Costello EJ Messer SC Pickles A Development of a short questionnaire for use in epidemiological studies of depression in children and adolescents Int J Methods Psychiatr Res 1995 5 237 249

[B33] Fairclough DL Design and Analysis of Quality of Life Studies in Clinical Trials 2010 Boca Raton, FL: Chapman and Hall/CRC

[B34] Pinheiro J Bates D Mixed Effects Models in S and S-Plus 2000 New York: Springer-Verlag

[B35] Preacher KJ Hayes AF Asymptotic and resampling strategies for assessing and comparing indirect effects in multiple mediator models Behav Res Methods 2008 40 879 891 10.3758/BRM.40.3.879 18697684

[B36] MacKinnon DP Lockwood CM Hoffman JM West SG Sheets V A comparison of methods to test mediation and other intervening variable effects Psychol Methods 2002 7 83 104 11928892 10.1037/1082-989x.7.1.83 PMC2819363

[B37] Muthén BO Muthén LK Mplus User’s Guide 2010 6 Los Angeles, CA: Muthén & Muthén

[B38] Guo S Analyzing grouped data with hierarchical linear modeling Child Youth Serv Rev 2005 27 637 652 10.1016/j.childyouth.2004.11.017

[B39] Heinrich CJ Lynn LE Means and ends: A comparative study of empirical methods for investigating governance and performance J Public Admin Res Theory 2001 11 109 138 10.1093/oxfordjournals.jpart.a003490

[B40] Constantino MJ Arnkoff DB Glass CR Ametrano RM Smith JZ Expectations J Clin Psychol 2011 67 184 192 10.1002/jclp.20754 21128304

[B41] Devilly GJ Borkovec TD Psychometric properties of the credibility/expectancy questionnaire J Behav Ther Exp Psychiatry 2000 31 73 86 10.1016/S0005-7916(00)00012-4 11132119

[B42] Goossens ME Vlaeyen JW Hidding A Kole-Snijders A Evers SM Treatment expectancy affects the outcome of cognitive-behavioral interventions in chronic pain Clin J Pain 2005 21 18 26 10.1097/00002508-200501000-00003 15599128

[B43] La Greca AM Silverman WK Lochman JE Moving beyond efficacy and effectiveness in child and adolescent intervention research J Consult Clin Psychol 2009 77 373 382 19485580 10.1037/a0015954

[B44] Smith P Yule W Perrin S Tranah T Dalgleish T Clark D Cognitive behavior therapy for PTSD in children and adolescents: a preliminary randomized controlled trial J Am Acad Child Adolesc Psychiatry 2007 46 1051 1061 10.1097/CHI.0b013e318067e288 17667483

[B45] Ehlers A Clark DM A cognitive model of posstraumatic stress disorder Behav Res Ther 2000 38 319 345 10.1016/S0005-7967(99)00123-0 10761279

